# Neutralizing Antibodies Limit Cell-Associated Spread of Human Cytomegalovirus in Epithelial Cells and Fibroblasts

**DOI:** 10.3390/v14020284

**Published:** 2022-01-28

**Authors:** Nina Reuter, Barbara Kropff, William J. Britt, Michael Mach, Marco Thomas

**Affiliations:** 1 Virologisches Institut, Klinische und Molekulare Virologie, Friedrich-Alexander-Universität Erlangen-Nürnberg, 91054 Erlangen, Germany; nina.reuter@uk-erlangen.de (N.R.); barbara.kropff@uk-erlangen.de (B.K.); michael.mach@fau.de (M.M.); 2 Departments of Pediatrics, Microbiology and Neurobiology, School of Medicine, Children’s Hospital of Alabama, University of Alabama, Birmingham, AL 35294, USA; WBritt@uabmc.edu

**Keywords:** herpesviruses, human cytomegalovirus (HCMV), glycoproteins, antibodies, neutralization, cell-to-cell spread, cell-associated spread, plaque size reduction

## Abstract

Human cytomegalovirus (HCMV) can cause severe clinical disease in immunocompromised individuals, such as allograft recipients and infants infected in utero. Neutralizing activity of antibodies, measured as the ability to prevent the entry of cell-free virus, has been correlated with the reduction in HCMV transmission and the severity of HCMV-associated disease. However, in vivo HCMV amplification may occur mainly via cell-to-cell spread. Thus, quantifying the inhibition of cell-to-cell transmission could be important in the evaluation of therapeutic antibodies and/or humoral responses to infection or immunization. Here, we established a quantitative plaque reduction assay, which allowed for the measurement of the capacity of antibodies to limit HCMV spread in vitro. Using an automated fluorescence spot reader, infection progression was assayed by the expansion of viral plaques during the course of infection with various GFP-expressing viruses. We found that in contrast to non-neutralizing monoclonal antibodies (mAbs), neutralizing mAbs against both glycoprotein B and H (gB and gH) could significantly inhibit viral plaque expansion of different HCMV strains and was equally efficient in fibroblasts as in epithelial cells. In contrast, an anti-pentamer mAb was active only in epithelial cells. Taken together, our data demonstrate that specific anti-HCMV mAbs can significantly limit cell-associated virus spread in vitro.

## 1. Introduction

Human cytomegalovirus (HCMV) is a ubiquitous betaherpesvirus with a 60–90% seroprevalence rate in the adult human population [1]. While HCMV infection in immunocompetent hosts is usually clinically asymptomatic, severe disease can result from primary infection or viral reactivation from latency in immunocompromised individuals. HCMV infects one out of every 150 live-born infants worldwide and therefore represents the most frequent cause of congenital viral infection [2]. HCMV is the leading infectious cause of childhood sensorineural hearing loss and a major cause of neurodevelopmental disorders [3]. In addition, HCMV is a major cause of morbidity and mortality in patients undergoing solid organ or stem cell transplantation [4,5]. Currently, antiviral drug therapeutic options are limited and their use can be restricted by their toxicity and the development of drug-resistant virus isolates [6]. Because of the clinical relevance, passive and/or active immunization strategies against HCMV are urgently needed and the development of a preventive vaccine has been given top priority by health authorities [7].

In the immunocompetent host, infection with HCMV is well controlled by a multifaceted immune response, including innate and adaptive immunity. As a component of the adaptive arm of the immune system, antiviral antibodies are thought to contribute significantly to the control of virus replication and spread. In the case of congenital HCMV infection, naturally acquired maternal antibodies have been considered to be important in limiting both the frequency and consequences of transplacental transmission of the virus [8]. In the transplant setting, high titers of HCMV-specific antibodies have been shown to be associated with an improved clinical outcome [9]. Although controversial, some studies have suggested that passive transfer of HCMV hyperimmune globulin (HIG) can be beneficial for the prevention and treatment of congenital HCMV infections and in certain transplant settings can ameliorate posttransplant HCMV disease [10,11,12,13,14,15,16,17]. More conclusive evidence for the protective role of antibodies in HCMV infection has been provided by animal studies. As demonstrated by Klenovsek et al., adoptive transfer of immune serum protected immunodeficient mice from the lethal course of murine CMV (MCMV) infection [18]. In addition, in infected newborn mice, antibodies provided protection from MCMV-induced pathology in the brain [19]. Finally, in a nonhuman primate model of congenital HCMV infection, preexisting antibodies were able to prevent severe congenital infection manifested by fetal loss [20]. Compared to polyclonal immunoglobulin preparations, monoclonal antibodies (mAbs) offer several advantages including improved consistency in manufacturing, greater potency, and reduced toxicity. Recently, passive transfer of an antiviral mAb conferred protection in the guinea pig model of congenital GPCMV infection [21]. Similarly, passive transfer of mAbs reactive with MCMV gB protected immune-compromised mice from MCMV infection [22]. Thus, antibodies with robust neutralizing capacity are in development for potential use as therapeutics or for passive prophylactic immunization (reviewed in [23,24]). Indeed, it was recently reported in high-risk renal transplant recipients that treatment with a combination of HCMV mAbs resulted in a decreased number of patients treated for HCMV disease and a delay in the interval to the development of viremia when compared to the placebo treatment group [25].

It is currently assumed that neutralizing antibodies that interfere in vitro with the envelope glycoprotein-mediated entry of the virus into the host cells likewise protect from HCMV infection in vivo [23]. Neutralizing targets of HCMV entry are complex and cell type-specific. Glycoprotein B represents the primary viral fusion protein which is essential for infection of all types of target cells [26]. Activation of gB’s fusogenic activity requires association with the gH/gL/gO complex which allows for viral entry into fibroblasts [27,28,29]. Infection of endothelial, epithelial, and dendritic cells requires an additional pentameric complex composed of gH/gL/UL128/UL130/UL131A [28,30,31,32,33,34]. Consequently, antibodies directed against gB or gH/gL exert their potent neutralizing activity independent from the cell type (reviewed in [35]). In contrast, neutralization by antibodies directed against the pentameric complex, in particular the proteins encoded by UL128-UL131A, was restricted to endothelial, epithelial, and dendritic cells with reduced activity on fibroblasts or trophoblast progenitor cells, the cell types which are crucial for the vertical HCMV transmission [36,37,38].

The role of HCMV neutralizing antibodies in the control of HCMV infection in vivo remains elusive since neutralization represents an in vitro measurement of the capacity of antibodies to reduce virus infectivity, which is typically determined by the inhibition of virus entry. However, in vivo HCMV is thought to disseminate primarily via direct cell-to-cell spread rather than through the release of cell-free virus [39,40]. In HCMV-infected individuals, the amount of extracellular virus detected by most assays is generally very low and most of the infectious virus in the blood is found in the leukocyte compartment, rather than in plasma or serum [41,42]. Moreover, some in vitro low-passage clinical HCMV isolates spread in a predominantly cell-associated manner and loss of this phenotype correlates with mutational changes in the viral genome [43,44,45]. These observations were confirmed in a recent study in which some HCMV clinical isolates spread almost exclusively cell-to-cell with cell-free spread being approximately three orders of magnitude less efficient than cell-to-cell spread [46]. Nevertheless, antibodies have an important impact on viral amplification. In the mouse model they have been shown to be able to limit HCMV dissemination and cell-associated spread [47,48]. While it is generally accepted that HCMV spread in vitro can be inhibited by antiviral antibodies in endothelial and epithelial cell cultures, whether antibodies can prevent subsequent rounds of infection that are mediated primarily by direct cell-to-cell transmission in cultured fibroblasts remains controversial [38,46,49,50,51,52,53,54,55,56,57,58].

It should be noted, however, that the term cell-to-cell spread is only operationally defined as the spread of the virus in fibroblast cultures resisting neutralization. The mechanism of this process has not been elucidated. In addition, whether the results using virus isolates which are restricted to replication via the cell-to-cell route reflects the situation found in vivo also remains to be determined as to the best of our knowledge unbiased studies analyzing a representative number of recent virus isolates with respect to their production of cell-free virus and resistance to antibody neutralization are lacking. In light of this imponderability, we have chosen for our study a more comprehensive but less specific term of “cell-associated virus spread” that defines the spread from an infected cell to adjacent cells under a semi-solid overlay containing virus-neutralizing antibodies.

To this end, we have developed an observer bias-independent automated quantification system for the systematic analysis of antibody-mediated inhibition of plaque formation. A diverse panel of well-characterized mAbs targeting gB, gH, and the pentamer complex were evaluated for their capacity to inhibit cell-associated HCMV spread. While non-neutralizing (nnt) antibodies failed to inhibit plaque formation, neutralizing (nt) antibodies limited cell-associated HCMV spread. Importantly, the inhibitory activity of a pentamer-specific mAb was restricted to epithelial cells, while all analyzed anti-gB and anti-gH mAbs limited cell-associated HCMV spread in HFF fibroblasts and ARPE-19 epithelial cells with comparable efficiency.

## 2. Materials and Methods

### 2.1. Cells and Viruses

Primary human foreskin fibroblasts (HFF) were prepared from human foreskin tissue (cell culture repository of M.M. laboratory) and grown in Dulbecco’s modified Eagle’s medium (DMEM) (Gibco, Thermo Fisher Scientific, Waltham, MA, USA) containing 10% fetal calf serum (FCS) (Sigma Aldrich, St. Louis, MO, USA), glutamine (100 μg/mL), and gentamicin (350 μg/mL). Human retinal pigmented epithelial cells (ARPE-19) were cultured in DMEM/F12 (Gibco, Thermo Fisher Scientific, Waltham, MA, USA) containing 10% fetal calf serum (FCS) (Sigma Aldrich, St. Louis, MO, USA), glutamine (100 μg/mL), and gentamicin (350 μg/mL).

GFP expressing HCMV strains Towne [59], TB40/E (TB40) [60], TB40/E-del pentamer, and TR5 (TR) [61] were propagated in HFF or ARPE-19 cells and viral titers were determined by titration of virus stocks and automated quantification of the amount of GFP-positive HFF cells at 72 hpi using a CTL Immunospot^®^S6 analyzer (Cellular Technology Limited, Bonn, Germany). The genetic integrity of the pentamer complex was confirmed by sequencing of the UL128-131 gene loci of ARPE-19 cells infected with TB40/E and TR- before the respective inoculum was used in the PRA assay.

### 2.2. Antibodies

The following antibodies were used (Table 1): the gB-specific human mAbs C23 (Ti23) [62], 1G2 [63], SM10 [63], SM5-1 [64], and SDZ 89-104 [65]; the mouse anti-gB mAbs 27-39 [66] and 27-287 [67]; the gH-specific human mAb MSL-109 [68]; the mouse anti-gH mAbs 6E3, 3C11, and 2B10 [69] as well as 19B12 and 13H2 (kindly provided by Christian Sinzger, Ulm, Germany); the mouse pentamer-specific mAb 4I22, recombinantly expressed based on patent application [37]; the gp120-specific (HIV env) antibody b12 (kindly provided by Klaus Überla, Erlangen, Germany). The murine and human isotype controls mIgG2a (clone C1.18.4) and hIgG1, were purchased from BioXCell and Squarix Biotechnology, respectively. The murine anti-myc mAb (clone 1-9E10.2) was purchased from ATCC.

### 2.3. Plaque Reduction Assay (PRA)

Either 1.0 × 10^4^ HFF or 1.2 × 10^4^ ARPE-19 cells seeded in 96-well plates were infected with 100 plaque-forming units (PFU) of several different HCMV strains (Towne, TB40/E, TB40/E-del pentamer, and TR5). At 24 hpi, the medium was replaced with an agarose overlay medium to prevent the extracellular spread of HCMV. The overlay was prepared as follows: 0.8% agarose maintained at 45 °C in a water bath was mixed 1:1 with 600 µL of room temperature 2x DMEM supplemented with 25% FCS, glutamine, gentamicin and containing the respective antibodies (100 µg/mL or dilutions thereof), small molecule anti-HCMV inhibitors (GCV, 40 µM; LMV, 4 µM) or bovine serum albumin (BSA, 100 µg/mL). Starting at 4 dpi, the 96-well plates were imaged each day with a CTL Immunospot^®^S6 Ultimate UV Analyzer (Cellular Technology Limited, Bonn, Germany) for up to 18–21 dpi. In the next step, two parameters were evaluated for each well separately over the time course of the infection experiment using the ImmunoSpot 6.0.0.2 software version (Cellular Technology Limited, Bonn, Germany): (i) the amount of single fluorescent spots/well was counted, confirming infection with an identical viral multiplicity of infections. This parameter should stay relatively constant over the entire infection experiment to exclude the coalescence of plaques. (ii) The size of all GFP-positive spots, which presumably represent plaques, was quantified for each well and time point. Based on the calculated different fluorescent spot sizes/well, the mean plaque size of each well was defined (ImmunoSpot 6.0.0.2 software), to monitor the size expansion of the viral plaques during the course of infection in the presence or absence of anti-HCMV antibodies. For direct comparison of the data obtained for different target cell types of HCMV, the PRA results were expressed as % reduction of mean plaque size relative to the no inhibitor control (0% inhibition) after subtraction of the initial fluorescent spot size, determined as mean spot size with no inhibitor measured at 4 dpi of the respective experiment.

### 2.4. Statistical Analysis

Statistical analysis was performed by ordinary one-way analysis of variance (ANOVA) using GraphPad Prism (version 6; GraphPad Software, San Diego, CA, USA).

## 3. Results

### 3.1. Establishment of a Live Cell Plaque Reduction Assay (PRA) for Quantitative Measurement of HCMV Spread

**Figure 1:**
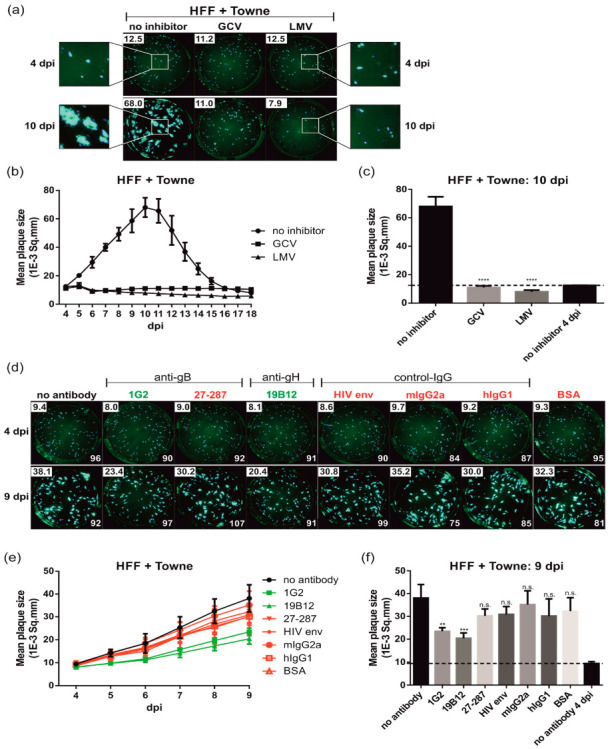
Plaque reduction assay (PRA) for automated quantification of cell–associated HCMV spread. HFFs in 96-well plates were infected with 100 PFU/well of HCMV strain Towne. After incubation for 24 h, the medium was replaced by agarose overlay medium containing the indicated (**a**–**c**) inhibitors (20 µM GCV, 2 µM LMV) or (**d**–**f**) antibodies (50 µg/mL) or BSA (50 µg/mL), respectively. Starting at 4 dpi, images of the whole 96-well were captured by a Fluorospot reader each day for at least 18 dpi and used for automated quantification of the mean plaque size (1E-3 Sq.mm) of all fluorescent spots detected per well as described in Materials and Methods. All experiments were performed in triplicate. (**a**,**d**) Representative images taken by the Fluorospot reader at the indicated dpi following treatment of HCMV-infected HFFs with the (**a**) HCMV inhibitors or (**d**) antibodies. (**a**) The magnifications demonstrate the size expansion of individual plaques over the course of the infection (4 dpi versus 10 dpi). (**a**,**d**) The numbers in the upper left corner represent the calculated mean plaque sizes (1E-3 Sq.mm) of the corresponding wells. (**d**) In the lower right corner, the total number of fluorescent spot counts is indicated. (**b**,**e**) Time course analyses of the mean plaque sizes of HCMV-infected cells treated with or without (**b**) HCMV inhibitors or (**e**) antibodies. nt mAbs are colored in green, nnt and negative control mAbs are shown in red. (**c**,**f**) Bar graphs illustrating the mean plaque sizes at the time points post infection at which the maximum mean plaque size of (**c**) the inhibitor control (10 dpi) or (**f**) no antibody control (9 dpi) was reached. The dashed lines indicate the mean plaque sizes of the (**c**) no inhibitor or (**f**) no antibody controls at 4 dpi, which reflects the initial fluorescent spot size of a single round infection. Statistical analysis was performed by ordinary one-way analysis of variance (ANOVA). n.s., not significant; **, *p*  ≤  0.01; ***, *p*  ≤  0.001; ****, *p*  ≤ 0.0001; *p* values refer to antibodies vs. no antibody control.

The capacity of antibodies to limit HCMV transmission in fibroblasts remains controversial. Discrepant results in the literature prompted us to establish a live cell PRA to investigate the impact of antibodies on HCMV spread within cell monolayers over time. Viral plaque formation was followed during the course of infection using a fluorescence spot reader (FluoroSpot reader) and GFP as a reporter. In this assay, HFF cells seeded in 96-well plates were infected with 100 PFU/well of GFP-expressing HCMV strain Towne and plaque formation quantified (Figure 1). In order to limit infection of adjacent cells via the extracellular release of the virus, the viral inoculum was replaced 24 hpi with an agarose overlay medium. Starting at 4 dpi, viral plaque size was monitored daily up to 18 dpi. The spot size of all individual fluorescent spots was measured automatically to calculate the mean plaque size per well as described in more detail in the Materials and Methods section. Initially, we determined the consistency of results provided by the assay by determining the effect of HCMV small molecule inhibitors on viral plaque progression (Figure 1a–c). At 24 hpi, together with the agarose overlay medium the DNA replication inhibitor ganciclovir (GCV) or the terminase inhibitor letermovir (LMV) were added to inhibit subsequent rounds of infection. In Figure 1a, sample images are shown from 4 and 10 dpi. As demonstrated by the inserts of Figure 1a, in the absence of inhibitor, viral plaques expanded in size as infection progressed (Figure 1a, compare 4 dpi and 10 dpi insert). As indicated in the upper left corner of the images in Figure 1a, the mean plaque size of the no inhibitor control increased from 12.5 1E-3 Sq.mm at 4 dpi to 68.0 1E-3 Sq.mm at 10 dpi. As expected, treatment with the HCMV replication inhibitors significantly limited the area of the plaque (Figure 1a). As shown in the time-course analysis, the mean plaque area remained constant over the duration of the experiment in the presence of GCV or LMV (Figure 1b). Furthermore, the time-course analysis revealed that the mean plaque size of the no inhibitor control increased steadily until 10 dpi where it reached its maximum after which it declined (Figure 1b). This decrease in plaque size was a consequence of the lysis of initially infected cells and loss of the corresponding GFP signals within plaques, which prevented the software from correctly quantifying individual plaques after several rounds of infection. This result clearly indicated the importance of defining the correct time point following infection when evaluating the potential effect of antibodies on HCMV spread, especially in regard to varying growth kinetics of different virus strains as well as cell types used for infection. Consequently, for all subsequent experiments, we chose the time point of maximum mean plaque size of the mock control to address the impact of inhibitors or antibodies on cell-associated HCMV spread (Figure 1c,f). Another critical experimental parameter that was established was the definition of the mean fluorescent spot size that developed even in cases in which the virus does not spread such as after treatment with GCV or LMV. This measurement represented the initial fluorescence signal that followed the first round of infection, and which was detectable regardless of treatments. Importantly, in accordance with the 96-h replication cycle of HCMV, on day 4 post infection the mean plaque size was still identical in all samples and matched the inhibitor treated samples throughout the duration of the experiment (Figure 1a–c). Thus, for all subsequent experiments the mean plaque size of the mock control at 4 dpi was used to define the baseline fluorescence signal that followed the first round of infection. This was indicated with a dashed line (Figure 1c,f). Next, the PRA was tested by using selected anti-HCMV antibodies directed against glycoproteins B (anti-gB; 1G2 and 27-287) and H (anti-gH; 19B12) along with a panel of negative control mAbs (control-IgG) including the gp120-specific antibody b12 (HIV env) or the murine and human isotype controls mIgG2a and hIgG1, respectively (Table 1; Figure 1d–f). HFFs were infected with Towne-GFP and the respective IgGs as well as a bovine serum albumin (BSA) control were added 24 hpi together with the agarose overlay medium. Viral plaque size expansion was recorded daily up to 21 dpi. Representative images of 4 dpi and 9 dpi are shown in Figure 1d. In addition, the assay was controlled by measuring the total number of fluorescent spots per well (Figure 1d). These measurements allowed us to confirm infection with identical plaque-forming units (Figure 1d, similar spot counts at 4 dpi), and controlled for the formation of new plaques or coalescence of adjacent plaques at later time points of infection which would otherwise lead to incorrect calculation of larger mean plaque sizes (Figure 1d, similar spot counts at 4 dpi compared to 9 dpi). As shown, the mean plaque size of all samples was similar at 4 dpi, while clear differences were detectable after subsequent rounds of replication (Figure 1d–f). In the presence of the anti-HCMV mAbs 1G2 (gB) and 19B12 (gH) plaque size progression was delayed, resulting in statistically significant reduced mean plaque sizes compared to the no antibody control at 9 dpi (Figure 1d–f). Importantly, this effect correlated with the neutralizing activity of 1G2 and 19B12, as neither the non-neutralizing anti-gB mAb 27-287 nor any of the negative controls significantly affected plaque formation (Figure 1d–f). Of note, similar results were obtained when this assay was performed in parallel without agarose overlay medium (data not shown). It can be assumed, that addition of 50 µg/mL of antibody (which corresponds to an approximately 250-fold excess to the IC50 of the mAbs) neutralized the cell-free virus completely. Nonetheless, an agarose overlay medium was used throughout our study in order to evaluate the cell-associated spread of the negative control without antibodies. Taken together, we established an experimental system that allowed the analysis of the kinetics of plaque size expansion after HCMV infection using an automated reader, thus largely eliminating observer bias.

### 3.2. Neutralizing Antibodies to gB and gH Significantly Inhibit HCMV Spread in Fibroblasts

**Figure 2:**
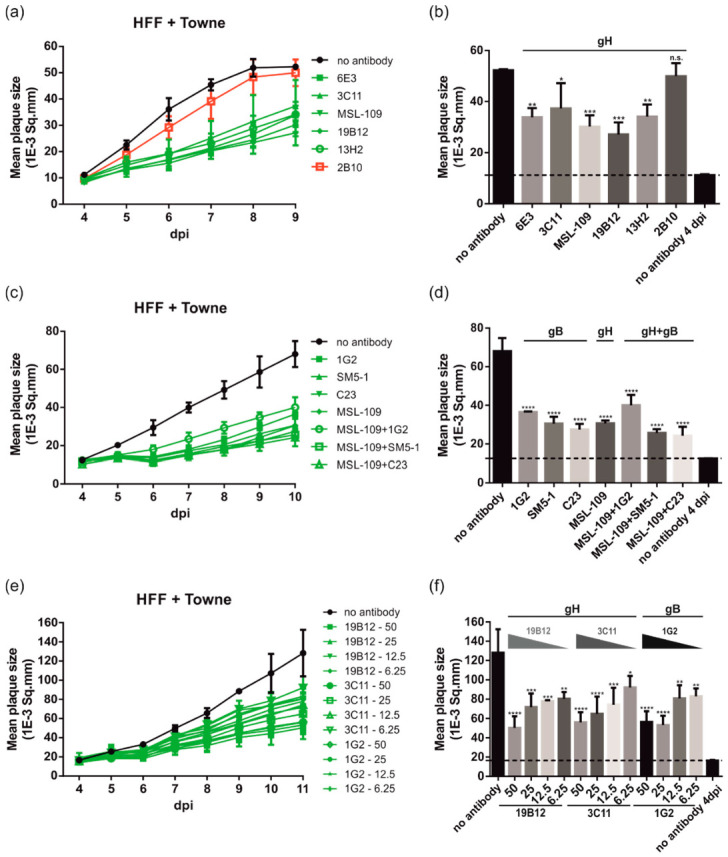
HCMV spread in fibroblasts is sensitive to inhibition by anti-gB and anti-gH antibodies. HFFs in 96-well plates were infected with 100 PFU/well of HCMV strain Towne. After incubation for 24 h, the medium was replaced by agarose overlay medium with or without the indicated (**a**,**b**) anti-gH antibodies (50 µg/mL), (**c**,**d**) anti-gB antibodies (50 µg/mL), anti-gB and anti-gH antibody combinations (25 µg of each antibody to a final concentration of 50 µg/mL), or (**e**,**f**) anti-gB and anti-gH antibody dilutions (50 µg, 25 µg, 12.5 µg, and 6.25 µg), respectively. Starting at 4 dpi, whole 96-well images were captured each day for at least up to 18 dpi and used for automated quantification of the mean plaque size (1E-3 Sq.mm) of all individual fluorescent spots detected per well as described in Materials and Methods. All experiments were performed in triplicate. (**a**,**c**,**e**) Time course analyses of the mean plaque sizes of HCMV-infected cells treated with or without (**a**) anti-gH antibodies, (**c**) anti-gB antibodies or anti-gB and anti-gH antibody combinations, or (**e**) anti-gB and anti-gH antibody dilutions. (**a**) The nt anti-gH mAbs are colored in green, the strain-specific anti-gH mAb 2B10 which does not neutralize Towne is shown in red. (**b**,**d**,**f**) Bar graphs of the mean plaque sizes at the time points post-infection with maximum mean plaque sizes of the no antibody controls ((**b**), 9 dpi; (**d**), 10 dpi; (**f**), 11 dpi). The dashed lines indicate the mean plaque sizes of the no antibody control at 4 dpi, illustrating the initial fluorescent spot size of a single-round infection. Statistical analysis was performed by ordinary one-way analysis of variance (ANOVA). n.s., not significant; *, *p*  ≤  0.1; **, *p*  ≤  0.01; ***, *p*  ≤  0.001; ****, *p*  ≤  0.0001; *p* values refer to antibodies vs. no antibody control.

**Figure 3:**
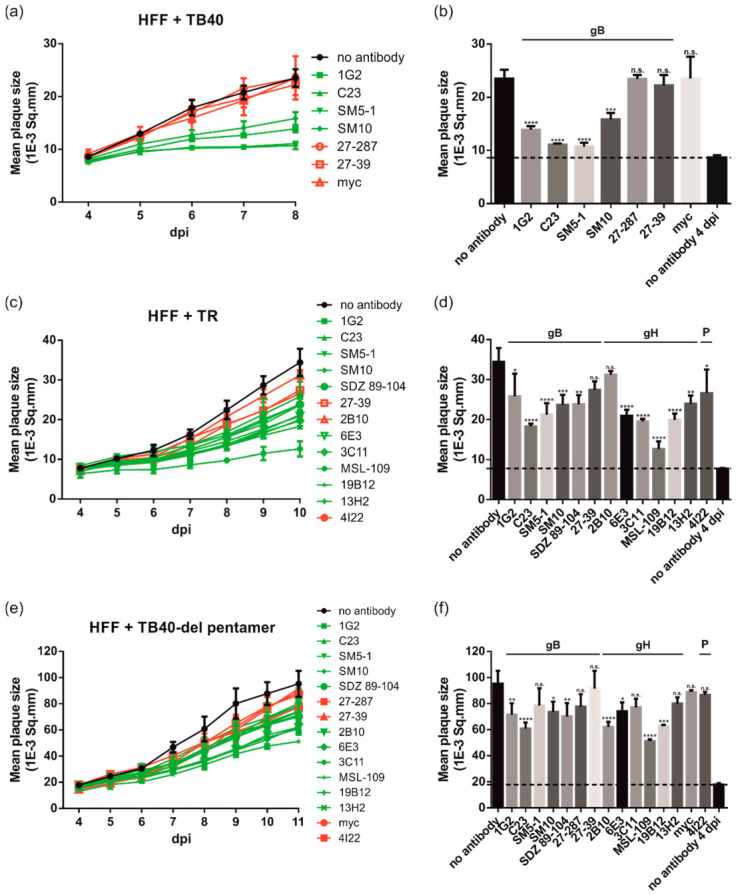
Anti-gH and -gB antibodies limit HCMV spread in fibroblasts in a strain-independent manner. HFFs in 96-well plates were infected with 100 PFU/well of different HCMV strains ((**a**,**b**) TB40/E; (**c**,**d**) TB40/E-del pentamer; (**e**,**f**) TR). After incubation for 24 h, the medium was replaced by agarose overlay medium with or without the indicated antibodies (50 µg/mL). Starting at 4 dpi, whole 96-well images were captured each day for at least up to 19 dpi and used for automated quantification of the mean plaque size (1E-3 Sq.mm) of all individual fluorescent spots detected per well as described in Materials and Methods. All experiments were performed in triplicate. (**a**,**c**,**e**) Time course analyses of the mean plaque sizes of HFF cells infected with the HCMV strains TB40/E (**a**), TB40/E-del pentamer (**e**), or TR (**c**). nt mAbs are colored in green, nnt and negative control mAbs are shown in red. (**b**,**d**,**f**) Bar graphs of the mean plaque sizes at the time points post-infection with maximum mean plaque sizes of the no antibody controls ((**b**), 9 dpi; (**d**), 10 dpi; (**f**), 11 dpi). The dashed lines indicate the mean plaque sizes of the no antibody controls at 4 dpi, which is indicative of the initial fluorescent spot size of a single-round infection. Statistical analysis was performed by ordinary one-way analysis of variance (ANOVA). n.s., not significant; *, *p*  ≤  0.1; **, *p*  ≤  0.01; ***, *p*  ≤  0.001; ****, *p*  ≤  0.0001; *p* values refer to antibodies vs. no antibody control.

After having established the assay, a broad panel of well-characterized neutralizing anti-gH (6E3, 3C11, MSL-109, 19B12, 13H2, and 2B10) and anti-gB mAbs (1G2, SM5-1, and C23) listed in Table 1 were assayed to address their impact on HCMV transmission in fibroblasts. As illustrated, all neutralizing antibodies tested limited HCMV spread significantly resulting in a 50–79% reduction of the mean plaque size at 9 and 10 dpi, respectively (Figure 2a–d). A notable exception was mAb 2B10 (9% reduction) which represents a potent strain-specific neutralizing antibody directed against gH of a subset of HCMV strains including TB40/E (Figure 3e) but not Towne (Figure 2a) or TR (Figure 3c) (Table 1) [69].

Next, we tested whether more effective reduction could be achieved by antibody combinations (Figure 2c,d). In this experiment, the anti-gH mAb MSL-109 was combined with anti-gB mAbs targeting different antigenic domains (AD) of gB (1G2: AD-5, SM5-1: AD-4, and C23: AD-2) (Figure 2c,d). None of the tested antibody combinations resulted in a significantly improved inhibition of plaque formation. Finally, serial dilutions (50–6.25 µg/mL) of selected anti-gH (19B12 and 3C11) and anti-gB (1G2) mAbs with cell spread inhibitory capacity were assayed (Figure 2e,f). This experiment demonstrated a concentration-dependent inhibitory effect of the antibodies (Figure 2e,f). Even antibody concentrations as low as 6.25 µg/mL could significantly limit cell-associated transmission of HCMV and reduced the mean plaque size up to 30–40% (Figure 2e,f). Nonetheless, 50 µg/mL of mAbs was used in the assay to exclude the spread of HCMV by the extracellular route through neutralization of free virus via an excess of antibody in the cell culture overlay medium [72]. Taken together, the data presented in Figure 1 and Figure 2 indicate that neutralizing anti-gB and anti-gH mAbs have the capacity to significantly inhibit cell-associated HCMV spread in fibroblasts.

### 3.3. gB and gH mAbs Limit Spread of Diverse HCMV Strains in Fibroblasts

Previous reports noted differences between HCMV strains with respect to inhibition of viral spread by antibodies [46]. To evaluate the effect of strain differences on the cell spread inhibitory activities of anti-gB and anti-gH mAbs in fibroblasts, the PRA was used together with viruses derived from TB40/E and the low-passage clinical HCMV strain TR (Figure 3). For some of the experiments, an extended set of antibodies including additional anti-gB mAbs (SM10, SDZ 89-104, and 27-39), the pentamer-specific mAb 4I22, and an anti-myc antibody as an additional negative control were used in the PRA (Table 1). As demonstrated by the time course analyses of TB40/E (TB40; Figure 3a) and TR (Figure 3c), plaque growth was reduced for both viral strains in the presence of mAbs with neutralizing activity as compared to non-neutralizing antibodies with a statistically significant reduction in mean plaque sizes at 8 dpi (TB40; Figure 3b) and 10 dpi (TR; Figure 3d), respectively. While neutralizing mAbs decreased the mean plaque size between 32–86%, most of the non-neutralizing mAbs exhibited considerably less plaque size reduction (0–36%). As an additional control to ensure that the monitoring of plaque reduction reflected cell-associated spread inhibition rather than a block of infection of adjacent cells via extracellular virus, the experiment was repeated with a pentamer complex-deleted variant of HCMV strain TB40/E (TB40-del pentamer; Figure 3e,f). Expression of the pentameric complex has been shown to promote cell-associated spread [30]. Consequently, TB40-del pentamer should be more prone to antibody-mediated inhibition of plaque expansion if inhibition of cell-associated spread resulted from the inactivation of cell-free virus. However, the decrease in plaque size of TB40-del pentamer virus-infected cells following treatment with neutralizing antibodies (e.g., 1G2, SM5-1, and SM10) was generally less when compared to wild type TB40/E (Figure 3a,b vs. Figure 3e,f). In some cases, neutralizing mAbs (SM5-1, 3C11, and 13H2) failed to significantly reduce the mean plaque size in TB40/E-del pentamer virus-infected cells (Figure 3f). In the presence of the pentamer complex (TB40/E; Figure 3a,b) neutralizing mAbs induced a plaque reduction of 52–86% compared to between 19–57% in the case of TB40-del pentamer (Figure 3e,f). These observations further strengthen the argument that neutralization of cell-free virus was not the mechanism of neutralizing antibodies in these assays of cell-associated virus spread in fibroblasts. As pentamer-specific mAbs are known to fail to neutralize HCMV infection in fibroblasts [37,38], 4I22 had no significant effect on cell-associated virus spread in this assay (Figure 3c–f). Of note, deletion of the pentamer increased plaque size without antibody about two-fold (Figure 3a,e, compare plaque sizes at 8 dpi), so the cell-associated spread is clearly enhanced by this mutation and the mutation somehow contributes to sensitivity to neutralizing antibodies. Overall, these data led to the conclusion that HCMV spread inhibition by gB- and gH-specific antibodies with virus-neutralizing activities was virus strain-independent in fibroblasts.

### 3.4. gB and gH mAbs Limit HCMV Spread Efficiently in Both Fibroblasts and Epithelial Cells

**Figure 4:**
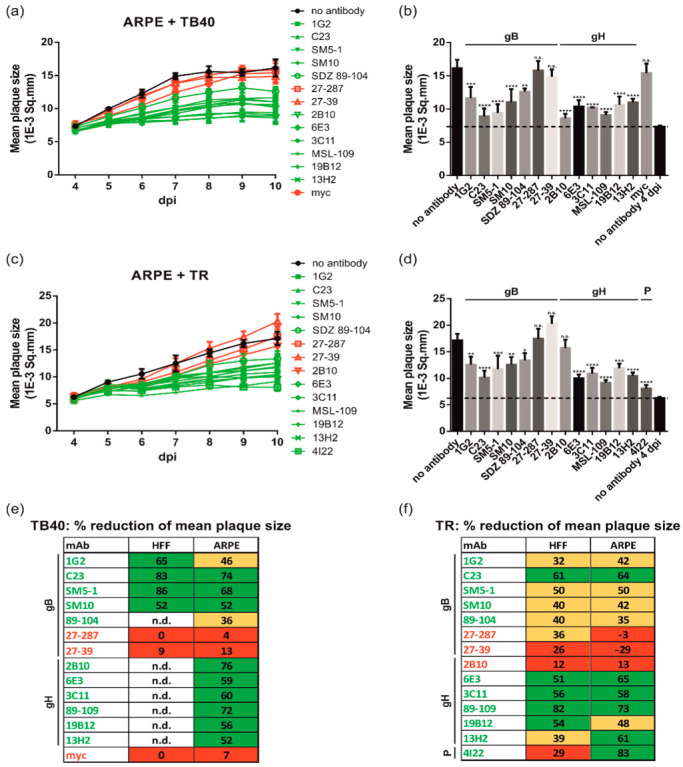
Anti-gB and anti-gH mAbs are equally efficient in limiting HCMV spread in fibroblasts and epithelial cells. ARPE-19 (ARPE) cells seeded in 96-well plates were infected with 100 PFU/well of HCMV strains (**a**,**b**) TB40/E and (**c**,**d**) TR, respectively. After incubation for 24 h, the medium was replaced by agarose overlay medium with or without the indicated antibodies (50 µg/mL). Starting at 4 dpi, whole 96-well images were captured each day for at least up to 21 dpi and used for automated quantification of the mean plaque size (1E-3 Sq.mm) of all individual fluorescent spots detected per well as described in Materials and Methods. All experiments were performed in triplicate. (**a**,**c**) Time course analyses of the mean plaque sizes of ARPE cells infected with the HCMV strains TB40/E (**a**) or TR (**c**). nt mAbs are colored in green, nnt and negative control mAbs are shown in red. (**b**,**d**) Bar graphs illustrating the mean plaque sizes at the time point of maximum mean plaque size of the no antibody controls (10 dpi). The dashed lines indicate the mean plaque sizes of the no antibody control at 4 dpi, which is indicative of the initial fluorescent spot size of a single-round infection. Statistical analysis was performed by ordinary one-way analysis of variance (ANOVA). n.s., not significant; *, *p*  ≤  0.1; **, *p*  ≤  0.01; ***, *p*  ≤  0.001; ****, *p*  ≤  0.0001; *p* values refer to antibodies vs. no antibody control. (**e**,**f**) Comparison of the capacity of the indicated mAbs to reduce the mean plaque size (given in % relative to the no antibody control after subtraction of the basic fluorescence mean spot size = mean plaque size of the no antibody control at 4 dpi) following infection of HFF as well as ARPE cells with TB40/E (**e**) and TR (**f**), respectively. Red, no significant reduction in mean plaque size (<30%); yellow, significant reduction in mean plaque size (30–49%); green, highly significant reduction in mean plaque size (>50%); n.d., not determined.

It has been described that HCMV cell-to-cell transfer is sensitive to antibody-mediated inhibition in epithelial and endothelial cell cultures [49,58]. For comparison with the data collected in fibroblasts, similar assays were conducted in ARPE-19 cells which were infected with 100 PFU of the epitheliotropic viruses TB40 and TR, respectively (Figure 4). Limited plaque size expansion was seen in the presence of neutralizing anti-gB and anti-gH mAbs (TB40, Figure 4a,b; TR, Figure 4c,d). This resulted in a similar grouping of neutralizing mAbs versus non-neutralizing mAbs in the time course analyses (TB40, Figure 4a; TR, Figure 4c) and decreased mean plaque sizes at 10 dpi (TB40, Figure 4b; TR, Figure 4d). The inhibitory capacity of neutralizing mAbs ranged between 35–83% as compared to a maximum of only 13% for the non-neutralizing control antibodies. For direct comparison of the results of the PRAs performed in fibroblasts and epithelial cells, the calculated % reduction of the mean plaque size in the presence of the individual antibodies is shown (Figure 4e,f). These results argued that all neutralizing mAbs tested were equally efficient in limiting cell-associated HCMV spread in fibroblasts as in epithelial cells following infection with TB40 (Figure 4e) and TR (Figure 4f), respectively. The only exception was 4I22 (Figure 4f), again confirming that pentamer-specific antibodies can only limit viral cell transmission in epithelial cells but not fibroblasts (Figure 4f). In summary, our data indicate that neutralizing anti-gB and anti-gH mAbs are capable of blocking HCMV cell transmission in fibroblast and epithelial cells with comparable activity.

## 4. Discussion

The exact mechanism(s) of HCMV amplification and dissemination in vivo is still unknown [40]. Replication of low passage HCMV isolates in vitro is highly cell-associated [43]. This suggests that in vivo HCMV amplification may occur mainly via spread between adjacent cells within tissues [43,73]. Therefore, antibody inhibition of HCMV spread may be more clinically relevant than neutralization of cell-free virus, thus assays to quantitate spread prevention may be more informative for vaccine and immunotherapeutic development. Therefore, we established a quantitative PRA for live cell imaging applications using GFP expressing virus as a reporter. Various measures were taken to ensure that monitoring of plaque formation reflected HCMV spread by the cell–cell route and not via extracellular release of the virus: (i) cells were overlayed with agarose containing medium in adaptation to the protocol of Jacob and colleagues, who validated their assay extensively to exclude any contribution of the extracellular virus to the number and size of plaques under these experimental conditions [55]. (ii) In all experiments an excess of antibody was added to the cell culture overlay medium to guarantee complete neutralization of potentially released cell-free virus [72]. (iii) Different HCMV strains were tested including the low passage clinical isolate TR (Figure 3 and Figure 4), which was stabilized in the form of a bacterial artificial chromosome after very limited passage in the fibroblasts [74]. (iv) Experiments were performed in the presence and absence of the pentamer complex (Figure 3, TB40 and TB40-del pentamer), which has been identified to facilitate spread via the cell–cell route [46], to further determine the contribution of extracellular virus release to the experimental outcome.

Our data demonstrate that mAbs against the essential viral glycoproteins B and H have the capacity to slow cell-associated HCMV spread. Interestingly and contrary to previous observations [38,75,76], we found that this inhibitory effect was independent of the target cell-type and the mAb concentrations as the respective anti-gB and anti-gH mAbs prevented HCMV spread equally efficiently in HFF and APRE-19 cells (Figure 4e,f). However, within the human host it seems possible that other cell types exist which may exhibit increased or reduced sensitivity to the mAbs that were tested in this study. Our finding argues that there is not a mechanistic difference between viral spread in either of these cell types and suggests that there is a common mode of virus spread between cells. All analyzed nnt mAbs were inactive in our PRA assays, while all tested nt mAbs significantly reduced spread in HFF and ARPE-19 cells, which is in accordance with previous findings in studies using epithelial cells [38]. We used high concentrations of mAbs in our assays (50µg/mL which is approximately 250-fold higher than the IC50 of the mAbs used in our studies). This is explained by the fact that infected cells presumably present a higher concentration of glycoproteins on the surface compared to viruses and importantly, cell-surface glycoproteins could continually deplete available antibodies due to endocytosis of glycoprotein/antibody complexes [77,78,79]. Relevant to the interpretation of our results from these in vitro studies are findings from studies that have used human mAbs in vivo [80]. Infusion of 50 mg/kg of an anti-gB mAb resulted in trough concentrations of 7.4 µg/mL in plasma, a concentration which in our assay showed inhibition of cell–cell spread in HFF (Figure 2f). Thus, although the biodistribution of mAbs in tissues may represent an important factor, it seems conceivable that following mAb therapy in vivo antibody concentrations could be achievable that are capable of inhibiting cell–cell spread. Furthermore, it should be noted that although diverse epitopes are targeted by the large panel of antibodies assayed in this study, it cannot be excluded that additional epitopes on gB or gH may exist that when targeted by antibodies can selectively function to inhibit viral entry or spread. The antibodies utilized in this study were identified by screening procedures that were based on antigen binding and/or virus neutralizing capacity, either of which can bias the identification of antibodies directed at a limited number of epitopes. In fact, in studies in mice (Reuter et al., in preparation), we found that immune sera from mice immunized with soluble recombinant gB that was used in human vaccination trials, efficiently blocked gB-induced fusion as measured in a recently established cell–cell fusion assay [81,82,83]. However, these sera lacked virus-neutralizing activity comparable to sera from vaccinated individuals [84,85,86]. Furthermore, using a murine CMV (MCMV) model, we could demonstrate that anti-gB mAbs were able to protect immunodeficient hosts from lethal infection even when administered therapeutically [22]. In contrast to the use of mAbs in a prophylaxis protocol, in this therapeutic protocol, antibodies likely target infected cells and limit viral dissemination by blocking cell–cell spread. In this previous study, we showed that neutralizing mAbs had a greater capacity to reduce the viral burden compared to non-neutralizing mAbs, which is consistent with our in vitro observation. Furthermore, in this same study mAbs with comparable in vitro virus-neutralizing capacities differed in their protective potency in vivo. Interestingly, we found that the in vivo protective capacity of anti-gB mAbs appeared to correlate with their ability to prevent cell-associated spread in vitro. Taken together, these findings further support the notion that antibody binding sites may exist on gB that allow selective targeting functions required for MCMV entry or spread and that targeting this specific function could limit dissemination and contribute significantly to the protective capacity of mAbs in vivo. Moreover, since the murine mAbs were found to bind to similar antigenic structures on MCMV gB that are present in HCMV gB, this finding suggests that human antibodies may exist which have the potential to entirely prevent HCMV spread, as none of the antibodies tested in the current study either administered alone or in different combinations was able to completely block plaque formation [22] (Figure 2c,d). This hypothesis is further supported by data from Gerna and co-workers, who identified an anti-gH mAb that effectively blocked plaque formation in both HUVECs (human umbilical vein endothelial cell) and HELFs (human embryonic lung fibroblasts) [58]. Our quantitative live cell PRA would allow for screening of antibodies that specifically block viral spread. Identifying their target epitopes could be important to improve the development of novel HCMV vaccines or therapeutic antibodies.

To further control our PRA, we included mAbs which either function in a virus strain-dependent (2B10) or cell-type-dependent manner (4I22). 2B10 is a novel mAb with a strict strain-specific epitope on gH, showing an “all or nothing” neutralization phenotype [69]. For instance, while it has a robust HCMV-neutralizing activity for TB40/E, it fails to neutralize strains like Towne or TR [69]. In the PRA, 2B10 only limited viral spread of TB40/E but not of Towne or TR (Figure 2, Figure 3 and Figure 4). On the contrary, 4I22 is a pentamer-specific antibody, which has potent neutralization capacity when assayed in epithelial and endothelial cells [37]. In our PRA, 4I22 was superior in the inhibition of epithelial cell spread compared to anti-gH or anti-gB mAbs. However, a major drawback of targeting only the pentameric complex is that antibodies against it fail to inhibit HCMV entry and cell-associated spread of fibroblasts (Figure 4f), whose ubiquitous distribution in the body may make them an important cell type for HCMV infection and amplification. The infection of fibroblasts is likely of major importance in transplant-associated HCMV disease, where viral spread within infected organs significantly contributes to HCMV pathogenesis.

Fluorescence-based assays for quantitative measurement of HCMV spread have been described previously [46,49,76]. However, these assays have not been utilized to: (i) compare the viral spread in different target cell types of HCMV, (ii) have been performed with an only limited number of antigen target specificities or, (iii) not in a live cell imaging setting to allow for monitoring of plaque expansion in a timely manner. Importantly, the correct timing of GFP measurement post-infection turned out to be of critical importance for optimal detection of antibody-mediated spread inhibition, particularly as these conditions were specific for each virus and cell type used for infection. Therefore, in this study, we carried out a series of experiments with GFP-reporter viruses in order to determine the optimal times for the PRA. Cloning of HCMV from clinical samples on bacterial artificial chromosomes (BACs) has resulted in a variety of genetically distinct strains for laboratory use. In our study, we included TR as a low-passage-number clinical isolate [74], as the TR-BAC is known to be mutationally stable in the UL128/UL130/UL131A pentameric gene loci when propagated in fibroblasts [87]. There may be viral strains that are characterized by an even more cell-associated spread phenotype [76]; however, it remains unclear how the genetic and phenotypic diversity of BAC-cloned strains reflect the natural diversity in vivo. There is growing evidence for a high genomic diversity of HCMV in the human host and that the observed adaptation in cell culture may represent a selection of preexisting genotypic variants rather than the acquisition of de novo mutations [43,88,89,90,91,92]. In our study, we included three different BAC-cloned viral strains (Towne, TB40/E, and TR) which importantly, were all inhibited by similar amounts when incubated with anti-gB and anti-gH antibodies in the PRA. We are aware of the fact that we have used a small number of strains that, in addition, may have undergone changes due to tissue culture adaptation and cloning. In vivo, HCMV strains may exist which may have increased or decreased sensitivity to the mAbs used in our study.

In summary, we established and comprehensively validated a quantitative live cell PRA to monitor HCMV spread via the cell–cell route. Utilizing this assay, we could demonstrate that in contrast to pentamer-specific mAbs, antibodies against the essential viral glycoproteins B and H are able to inhibit HCMV spread independent of the target cell-type as they were similarly effective in inhibiting viral dissemination in fibroblasts and epithelial cells. A better understanding of the target epitopes targeted by antibodies that block cell-associated HCMV transmission will likely be of great importance in the design of improved vaccine or therapeutic antibodies for prophylaxis and treatment of HCMV infections.

## Figures and Tables

**Table 1:** Anti-HCMV antibodies used in this study.

Antibody	Target	Immunogen	Species	Reference
Neutralizing				
1G2	gB/AD-5	Natural infection	Human	[63,64]
SM5-1	gB/AD-4	Natural infection	Human	[64,70,71]
SM10	gB/AD-5	Natural infection	Human	[63,64]
C23	gB/AD-2	Natural infection	Human	[62]
SDZ 89-104	gB/AD-1	Natural infection	Human	[65]
2B10	gH	AD169	Mouse	[69]
6E3	gH	AD169	Mouse	[69]
3C11	gH	AD169	Mouse	[69]
19B12	gH	TB40/E	Mouse	[unpub.]
13H2	gH	TB40/E	Mouse	[unpub.]
MSL-109	gH	Natural infection	Human	[68]
4I22	pUL128/130/131	Natural infection	Human	[37]
Non-neutralizing				
27-287	gB/AD-1	AD169	Mouse	[67]
27-39	gB/AD-1	AD169	Mouse	[66]

## Data Availability

Not applicable.
